# Phase-contrast imaging with synchrotron hard X-ray reveals the effect of icariin on bone tissue morphology and microstructure in rabbits with early glucocorticoid-induced osteonecrosis of the femoral head

**DOI:** 10.3389/fcell.2023.1155532

**Published:** 2023-05-04

**Authors:** Xu Yang, Lijun Shi, Aifeng Li, Fuqiang Gao, Wei Sun, Zirong Li

**Affiliations:** ^1^ Department of Orthopedics, Peking University China-Japan Friendship Clinical Hospital, Beijing, China; ^2^ Health Science Centre, Peking University, Beijing, China; ^3^ Department of Orthopedic Surgery, The First Affiliated Hospital of Zhengzhou University, Zhengzhou, Henan, China; ^4^ Department of Nephrology, Xiyuan Hospital, China Academy of Chinese Medical Sciences, Beijing, China; ^5^ Centre for Osteonecrosis and Joint-Preserving & Reconstruction, Orthopaedic Department, China-Japan Friendship Hospital, Beijing, China; ^6^ Department of Orthopaedic Surgery, Perelman School of Medicine, University of Pennsylvania, Philadelphia, PA, United States

**Keywords:** phase-contrast imaging with synchrotron hard X-ray, osteonecrosis of the femoral head (ONFH), glucocorticoid (GC), icariin (ICA), bone microstructure

## Abstract

**Background:** Phase-contrast imaging (PCI) with synchrotron hard X-ray was used to observe the changes in bone tissue morphology and microstructure in rabbit models of early glucocorticoid-induced osteonecrosis of the femoral head (ONFH), and to evaluate the intervention effect of Icariin.

**Methods:** Fifty mature New Zealand rabbits (weighing 2.5–3.0 kg) were randomly divided into a control group (*n* = 10), a glucocorticoid group (*n* = 20), and an Icariin group (*n* = 20). The glucocorticoid group and the Icariin group were sequentially injected with lipopolysaccharide (LPS) and methylprednisolone (MPS) to establish a glucocorticoid-induced ONFH animal model. The Icariin group was given Icariin solution when methylprednisolone was injected for the first time, and the control group and glucocorticoid group were given the same amount of normal saline. Animals were sacrificed after 6 weeks, and bilateral femoral head specimens were taken for research. The right femoral head was observed by PCI with synchrotron hard X-ray technology, and the left femoral head was verified by Micro-CT scanning and HE staining.

**Results:** Forty-three animals (nine in the control group, sixteen in the glucocorticoid group, and eighteen in the Icariin group) were included in the study. PCI with synchrotron hard X-ray revealed that the trabecular bone in the glucocorticoid group was thinned, broken, and structurally damaged, whereas the trabecular bone in the Icariin group had normal volume, thickness, and a relatively intact structure. Micro-CT scan reconstruction and HE staining were used to verify the reliability of this technique in identifying osteonecrosis.

**Conclusion:** The effects of Icariin were observed in an early glucocorticoid-induced ONFH rabbit model using PCI with synchrotron hard X-ray. Icariin weakens the destructive effect of glucocorticoids on bone tissue structure, improves bone tissue morphology, and stabilizes bone microstructure. This technique may provide a definitive, non-invasive alternative to histological examination for the diagnosis of early ONFH.

## 1 Introduction

Synchrotron radiation light source is a kind of electromagnetic radiation produced when charged particles moving close to the speed of light move along arc-shaped orbits in a magnetic field. It has the characteristics of high luminance, high collimation, and high spatial coherence (Johnston et al., 1996; Gao et al., 1998; Betz et al., 2007; Pfeiffer et al., 2008; Martin-Garcia et al., 2017). Phase-contrast imaging (PCI) is an imaging technique based on the phase shift distribution caused by the X-ray refraction effect, including interferometry, diffraction, and in-line holography (Sun et al., 2011). PCI is superior to conventional absorption imaging in terms of readability and resolution (Momose et al., 1996). It introduces the refraction effect of synchrotron radiation into the spatial phase shift distribution, can record different structural information in weakly absorbing substances, and display X-ray two-dimensional (photography) or three-dimensional (CT) microscopic images (Sun et al., 2011). The boundary density of different tissues varies between 0.0003–0.002 g/cm^3^, and the spatial resolution can reach 0.2 nm (Sun et al., 2015). The X-ray contrast resolution produced by the synchrotron radiation source is more than 1,000 times higher than the ordinary X-ray density resolution, which has great potential in the fields of life science and medicine.

Osteonecrosis of the femoral head (ONFH) is a common and refractory orthopedic disease, which occurs in young and middle-aged people, and has a high disability rate, seriously affecting the social work and quality of life of patients (Cohen-Rosenblum and Cui, 2019). According to the etiology, this disease can be divided into two categories: traumatic and non-traumatic. The pathological process and mechanism of the latter are very complex, and common risk factors include long-term or heavy use of glucocorticoids, alcoholism, decompression sickness, chronic kidney disease, blood diseases, organ transplantation, and autoimmune diseases (Cui et al., 2021). Glucocorticoids have become the first cause of nontraumatic ONFH and are by far the most common type of nontraumatic ONFH (Cooper, 2004), and some foreign scholars have reported that up to 51% of nontraumatic ONFH belong to this type (Fukushima et al., 2010). An estimated 20,000 to 30,000 new patients are diagnosed with osteonecrosis each year (Mankin, 1992; Aldridge and Urbaniak, 2004; Moya-Angeler et al., 2015). During the treatment of COVID-19 in Wuhan in 2020, the use of glucocorticoids was as high as 44.9% (Wang et al., 2020; Li et al., 2021a). Mont et al. concluded that ONFH occurs in approximately 58%–80% of patients within 3 years of starting glucocorticoids if they are not effectively treated (Mont et al., 2015). Therefore, regular screening of patients on long-term glucocorticoids is needed for early diagnosis and prompt treatment.

Icariin (ICA), an 8-prenylated flavonoid glycoside with the molecular formula ICA/C33H40O15, is the main active ingredient of the Chinese Herb Epimedii Folium, which is known for its “bone-strengthening” effects. Modern pharmacological studies have confirmed that Epimedium has various pharmacological effects such as regulating bone metabolism and promoting angiogenesis (Wu et al., 2017; Zhang et al., 2017; Wang et al., 2018). In the regulation of bone metabolism, ICA can not only promote osteoblast proliferation and differentiation to accelerate osteogenesis but also inhibit osteoclast activity and reduce bone resorption, thus positively regulating bone metabolism and improving bone quality (Liu et al., 2017; Tang et al., 2018; Wang et al., 2018). It has good efficacy in the treatment of bone diseases such as osteoporosis, osteoarthritis, and ONFH (Yu et al., 2019).

A multicenter randomized controlled clinical study led by our center previously showed that ICA was effective in preventing the occurrence of glucocorticoid-induced ONFH, reducing the incidence by 50% (Li et al., 2018). However, definitive, non-invasive alternatives to histological examination are currently lacking to further investigate the morphology, microstructure, and repair response of Icariin in glucocorticoid-induced ONFH. Because the anatomy of the femoral blood supply and the relative size of the femoral head are similar to those of humans, rabbits have become the animal model of choice for studying the pathogenesis and treatment of ONFH (Casey et al., 2021). We hypothesized that an early ONFH model was generated by sequential injections of lipopolysaccharide (LPS) and methylprednisolone (MPS) in New Zealand rabbits (Xu et al., 2018). PCI with synchrotron hard X-ray was used to observe the changes in bone tissue morphology and microstructure in rabbit models of early glucocorticoid-induced ONFH and to evaluate the intervention effect of ICA on early glucocorticoid-induced ONFH. And Micro-CT scan reconstruction, pathological detection, and other invasive mature technology were used for verification.

## 2 Materials and methods

### 2.1 Study design

Fifty adult New Zealand rabbits (2.5–3.0 kg, half male and half female)were purchased from Beijing Keyu Animal Breeding Center. All animals were housed in separate cages and kept under the same conditions in the animal laboratory of the China-Japan Friendship Hospital, with ration of feed and clean drinking water, room temperature of 20°C–25°C, humidity of about 50%, natural light, and good ventilation. This study was approved by the Ethics Committee of the China-Japan Friendship Hospital (2016-GZR-4) and conducted in accordance with the Committee’s guidelines. After 1 week of adaptive feeding, the experimental animals were randomly divided into three groups: the control group (*n* = 10), the glucocorticoid group (GC group; *n* = 20), and the Icariin group (ICA group; *n* = 20). No significant differences were found in body weight and sex composition ratio among the three groups (*p* > 0.05). The GC group and ICA group were injected with lipopolysaccharide (LPS; 5 μg/kg) and methylprednisolone (MPS; 20 mg/kg) sequentially in order to create an osteonecrosis of the femoral head (ONFH) model. After the first MPS injection, the ICA group was orally administered icariin solution (1 time/day for 6 weeks; calculated dose via experimental animal dose conversion) in 10 mL of distilled water. The control group and GC control group were given 10 mL of normal saline at corresponding time points for 6 weeks. All experimental animals were fed similarly and were forced to walk upright with their lower limbs via a suspension method. Furthermore, penicillin sodium (80,000U) was injected bilaterally into the gluteal muscles every week to prevent infection. Finally, the rabbits moved freely outside their cages for 2 h a day in order to increase activity and range of motion.

### 2.2 Observation indicators

The experimental animals were observed for survival, activity, mental status, diet, defecation, fur coloration, and the presence of infection on the body surface, and weighed weekly. Six weeks after the first injection of MPS, the experimental animals in the three groups were anesthetized with 20% ethyl carbamate intravenously and sacrificed. Dissect the hip joint, fully expose the femoral heads on both sides, and observe the contour of the femoral head, surface cartilage color, smoothness, and cartilage exfoliation. The right femoral head specimen was used for PCI with synchrotron hard X-ray, and the left femoral head specimen was used for Micro-CT scan reconstruction and HE staining. In this experiment, PCI with synchrotron hard X-ray was used to scan the bone tissue samples of each group, and the three-dimensional (3D) structure of bone trabecula in three groups were observed. At the same time, Micro-CT scanning reconstruction and HE staining were used to verify the reliability of this technique in identifying osteonecrosis.

**FIGURE 1:**
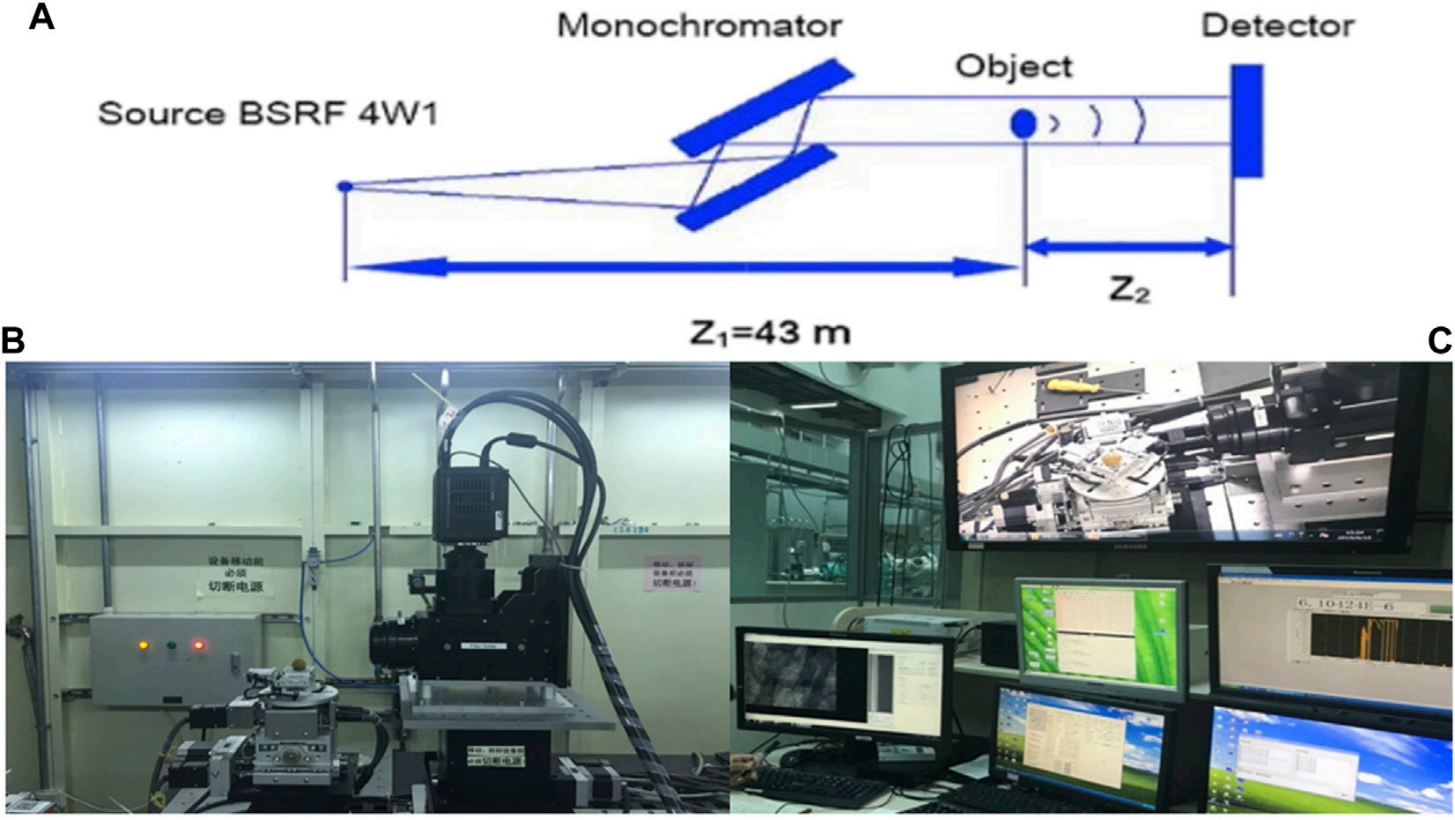
**(A)** Synchrotron radiation diagram. **(B)** Observation of femoral head specimens by phase-contrast imaging with synchrotron hard X-ray. **(C)** Synchrotron radiation imaging analysis of femoral head specimens.

As shown in Figure 1A, synchrotron radiation X-rays are refracted by two crystals and irradiated on the specimen, and the rays pass through the specimen and are imaged on the rear detector. The synchrotron radiation system used in this study comes from the Beamline Stations for X-ray Imaging and Biomedical Applications (BL13W1) of Shanghai Synchrotron Radiation Facility (SSRF). This station uses synchrotron broadband hard X-rays from a single-period electromagnetic oscillator plug-in as a light source in the energy range of 10–65 KeV. In this study, the photoelectron energy of synchrotron radiation is 2.2022 Gev, the critical energy of synchrotron radiation is 16 KeV, the exposure time is 0.5 s, the distance between the detector and the sample is 25 cm, and the resolution is 3.25 µm (Figure 1B).

Special surgical instruments such as trephine and small osteotome were used to obtain material from the right femoral head specimen and made a cylindrical specimen of 0.5 cm × 0.5 cm × 1 cm for the study. The imaging process of the study is as follows: X-rays emitted by synchrotron radiation pass through the experimental sample and hit the ray scintillation crystal plate behind. The scintillation film converts X-rays into visible light, and after being enlarged by the microscope objective lens group, it is projected onto the visible light CCD detector to collect visible light digital images and store them in BMP format. A visible light microscope and a visible light CCD are used as imaging detectors, and phase-contrast images are acquired in a large field-of-view mode. The rotation angle of the turntable is 0°–180°, the total number of projections is 2,000, and the scanning time is 30 min. The acquired image data is further processed by XMController software, and frame-by-frame images, animations, and tomographic images are obtained after 3D reconstruction (Figure 1C).

## 3 Results

A total of 7 experimental animals died during the study. One animal in the control group died of respiratory infection, one animal in the GC group died of acute diarrhea, three animals died after an intravenous injection of lipopolysaccharide (LPS), and two animals in the ICA group refused to eat after the first injection of LPS. Prednisolone (MPS) developed diarrhea and eventually died. At the end of the study, a total of 43 animals survived, including 9 in the control group, 16 in the GC group, and 18 in the ICA group. Except for the dead animals, all 9 animals in the GC group and ICA group showed varying degrees of unresponsiveness, depression, shortness of breath, and eyelid congestion after intravenous injection of LPS. Three animals developed diarrhea and loss of appetite, which gradually subsided after 24 h. After 3 consecutive injections of MPS, 10 animals showed decreased activity, unresponsiveness, diet, and weight loss, and returned to normal food intake after 4 weeks; the fur of animals in the GC group lacked luster. The animals in the control group had no obvious abnormality, moved freely, ate normally, gradually increased in body weight, and had smooth fur.

### 3.1 PCI with synchrotron hard X-ray

**FIGURE 2:**
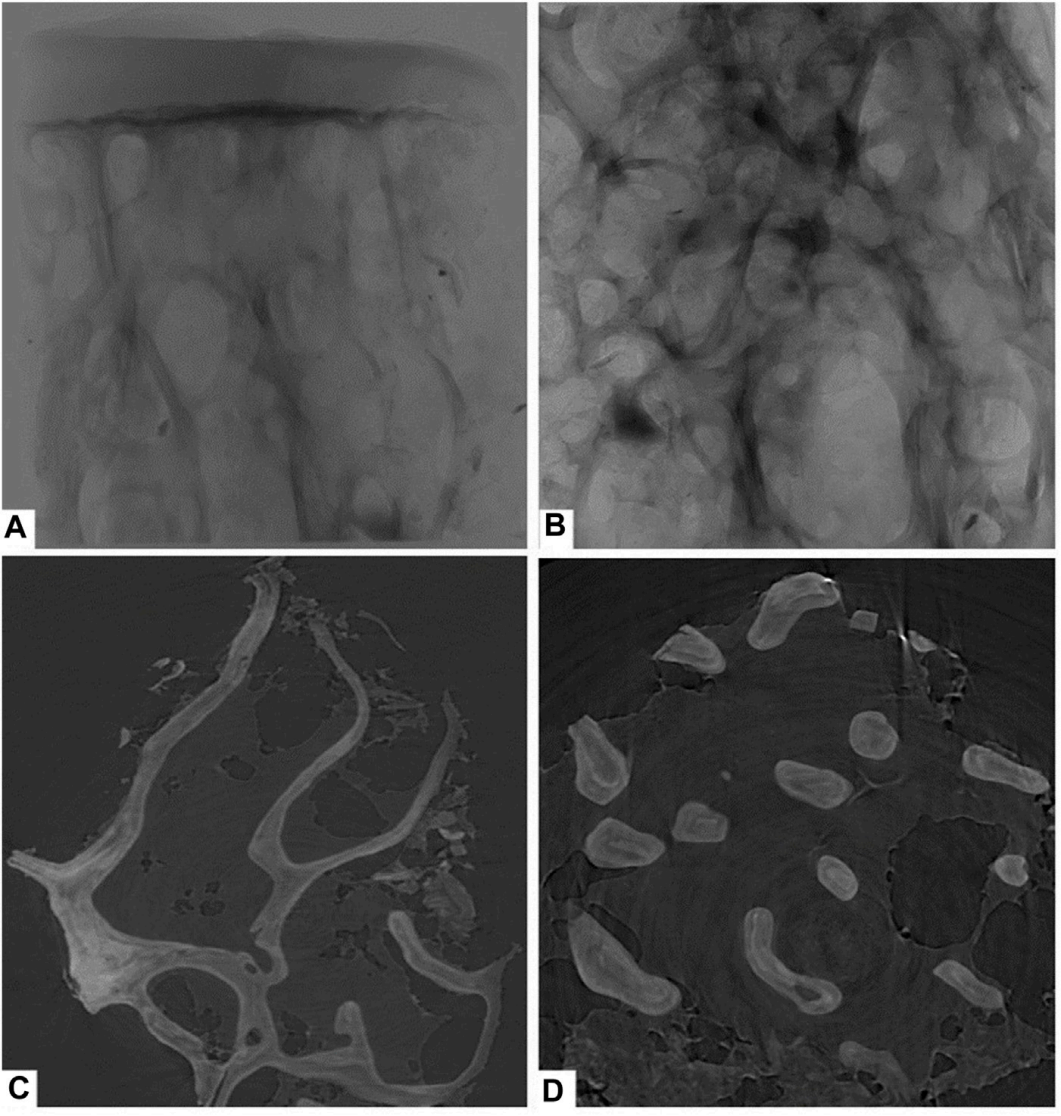
Phase-contrast imaging with synchrotron hard X-ray of the control group. **(A)** overall structure; **(B)** cancellous bone structure; **(C)** coronal reconstructions of trabecular bone; **(D)** cross-sectional reconstructions of trabecular bone.

This study used PCI with synchrotron hard X-ray to examine the right femoral heads of all groups. The PCI technique provided detailed images of the femoral head cartilage and trabecular bone. Figure 2A displays the results of the specimens in the control group, showing a smooth, intact, and defect-free cartilage surface. The subchondral bone plate was intact and the trabeculae were orderly and densely arranged. Figure 2B displays a structural image of cancellous bone, while Figures 2C, D present coronal and cross-sectional reconstructions of trabecular bone, respectively, which can be used to compare the amount of trabecular bone in the same field of view. The results of this study demonstrate the potential of the PCI with synchrotron hard X-ray technique to provide detailed images of the femoral head cartilage and trabecular bone, which can be used to assess the structure and morphology of these tissues.

**FIGURE 3:**
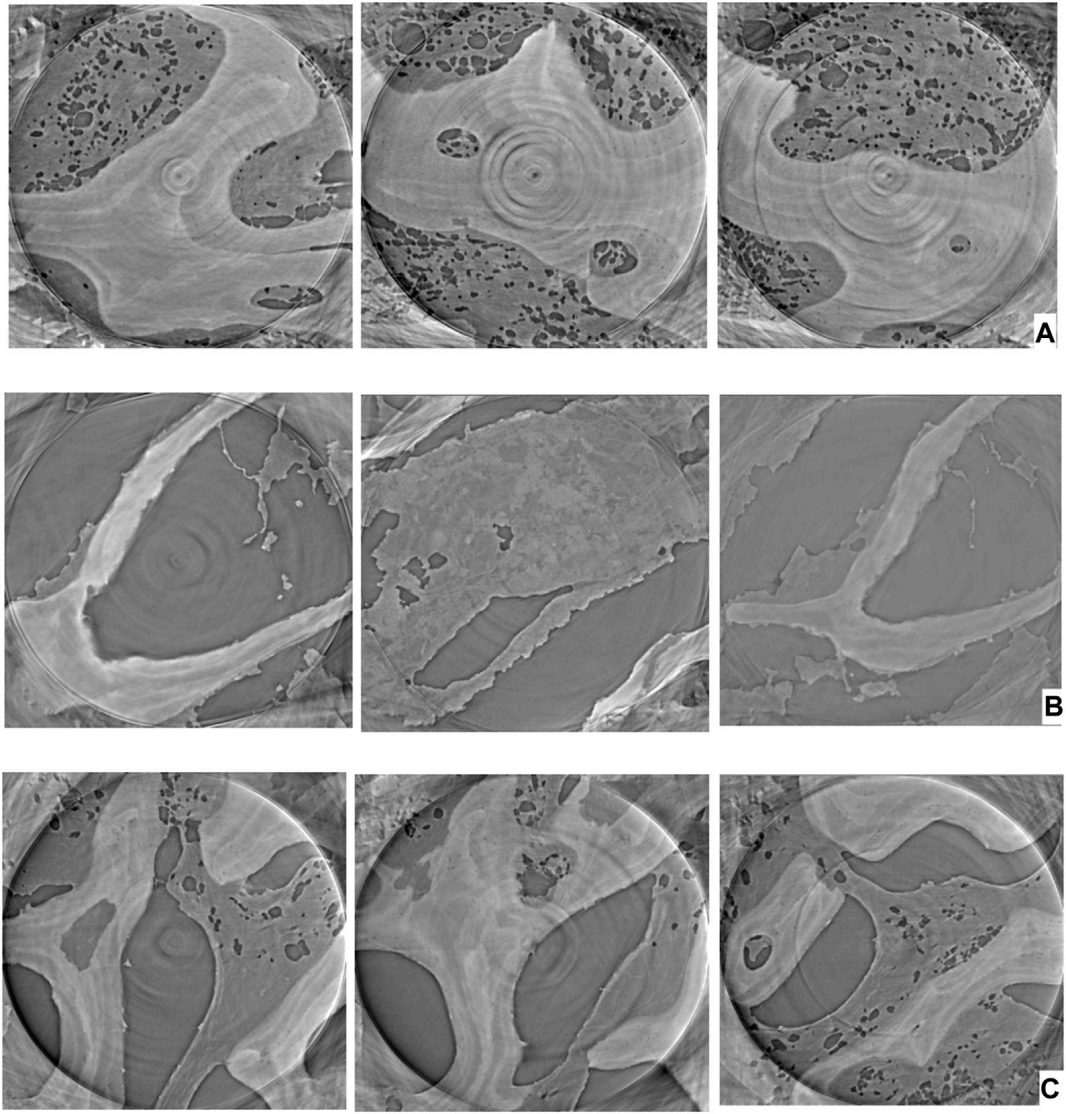
Ultrastructural imaging of bone tissue. **(A)** Control group; **(B)** GC group; **(C)** ICA Group.

Figure 3 shows the 3D imaging results of the ultrastructure of bone tissue specimens in each group. In the control group, the bone trabeculae were arranged neatly and continuously, with small gaps, thicker bone trabeculae, dense structure, smooth surface, complete shape, and no defect (Figure 3A). In the GC group, specimens of the femoral head with osteonecrosis revealed a disordered and fractured trabecular bone arrangement, a significantly widened gap, a reduced thickness of the trabecular bone, a reduced structural density, a rough surface, visible defects, and a severely damaged trabecular bone structure (Figure 3B). The results showed that, under the action of a high concentration of GC, osteocyte necrosis, apoptosis, and bone mass loss were severe, resulting in a serious disruption of the structural integrity of the bone trabecula. In the osteonecrosis femoral head specimens of the ICA group, the microstructure of the trabecular bone was slightly degenerated, yet the overall structural integrity was better than seen in the GC group. The trabecular bone was irregularly arranged, and its surface was rough, displaying defects in some areas (Figure 3C). The results showed that ICA had a protective effect on bone cell destruction, bone tissue morphology, and bone microstructure caused by GC, and could enhance the body’s ability to repair bone.

### 3.2 Verification

This study verified the reliability of PCI with synchrotron hard X-ray technique by bone histomorphology and HE staining on the left femoral head in all groups. Micro-CT scanning reconstruction and HE staining showed no ONFH in the control group. In the ONFH identified by the PCI with synchrotron hard X-ray technique in the GC group, both Micro-CT scan reconstruction and HE staining revealed thinning of the necrotic cartilage and fracture of the subchondral bone plate. The number of bone trabeculae in the femoral head decreased, the structure was disordered, the gap widened, the arrangement was sparse and irregular, and the continuity of some bone trabeculae was interrupted. In ONFH identified by the PCI with synchrotron hard X-ray technique in the ICA group, Micro-CT scan reconstruction and HE staining observed results similar to those in the GC group, but the extent of obvious lesions was milder. Only a few trabecular arrangements became sparse and a few trabecular structures were interrupted. These results demonstrate the reliability of the PCI with synchrotron hard X-ray technique to identify ONFH and its efficacy in assessing the severity of ONFH.

## 4 Discussion

*In vivo* imaging medical technology has developed from the X-ray photography technology discovered by Rontgen in 1895 to the CT technology discovered by Hounsfield at the end of the 20th century. X-ray and CT are difficult to find the early lesions of ONFH. At present, MRI is the main examination method for early diagnosis of ONFH, but it is expensive and time-consuming. In recent years, PCI with synchrotron hard X-ray technology has attracted more and more attention. Synchrotron radiation light source, as one of the four major artificial light sources, plays an important role in the fields of medicine and life science (Momose and Fukuda, 1995; Pisano et al., 2000; Takeda et al., 2000; Meuli et al., 2004), and a lot of research has also been carried out in the field of orthopedics. Synchrotron radiation imaging of trabecular bone was first reported by Salomé in 1999, followed by a series of other studies (Salomé et al., 1999). Weiss was the first to use synchrotron light to observe differences in bone cell growth in different biomaterials and obtain satisfactory images (Weiss et al., 2003). In previous studies, our team found that PCI with synchrotron hard X-ray can detect microscopic cartilage lesions that cannot be detected by traditional absorption contrast X-rays (Sun et al., 2015). In addition, in the rabbit ONFH model, the effectiveness of PCI on bone tissue repair was evaluated from the microscopic level (Sun et al., 2011).

The development of glucocorticoid-induced ONFH involves multiple mechanisms and is the result of the interaction of multiple pathogenic factors. Many theories about its mechanism include intravascular coagulation, abnormal differentiation of bone marrow mesenchymal stem cells, lipid metabolism disorder, inflammatory response, osteoporosis, and osteoclast apoptosis (Li et al., 2021b). The most widely accepted theory is lipid metabolism disorder: long-term or large-scale use of glucocorticoids leads to a significant increase in the concentration of glucocorticoids in the body, increased fat mobilization throughout the body, and tiny fat particles lead to bone microvascular embolism and local bone microcirculation disturbance. On the other hand, high concentrations of glucocorticoids decrease the osteogenic differentiation of bone marrow mesenchymal stem cells (BMSCs) and increase adipogenic differentiation, leading to fat accumulation in the bone marrow and increased intramedullary pressure of the femoral head. Exacerbate the reduction of local blood flow, bone cell ischemia, and hypoxia, and eventually cause osteonecrosis (Wang et al., 2000; Seamon et al., 2012; Mont et al., 2015; Shah et al., 2015). With the ongoing investigation of the pathogenesis of glucocorticoid-induced ONFH, a novel multiple-hit theory has been proposed. That is, high concentrations of glucocorticoids directly lead to the imbalance of bone homeostasis and cell apoptosis, and at the same time indirectly cause damage to the microcirculation in the bone by inhibiting the microcirculation in the new bone. The promotion of angiogenesis and blood hypercoagulability jointly lead to the occurrence of osteonecrosis (Seamon et al., 2012; Mont et al., 2015). Among them, the theory of osteocyte apoptosis and bone homeostasis imbalance has been paid more and more attention. Many studies believe that ONFH induced by glucocorticoids is a manifestation of massive apoptosis of bone cells, imbalance of bone homeostasis, and destruction of bone tissue morphology (Wang et al., 2019). On the one hand, glucocorticoids directly kill osteoblasts and osteocytes through endoplasmic reticulum stress and autophagy, resulting in increased apoptosis (Calder et al., 2004; Liu et al., 2018), decreased osteoblast activity and osteogenic activity, and decreased bone formation (Weinstein et al., 2000; Plotkin et al., 2007; Youm et al., 2010). On the other hand, glucocorticoids lead to enhanced bone resorption by osteoclasts in necrotic areas, leading to an imbalance in bone remodeling, which eventually leads to osteopenia, sparse trabecular bone, and decreased bone strength. When the trabecular bone structure in the necrotic area is subjected to a slight external force, microfractures will occur, and then the subchondral bone plate will fracture. As the disease progresses, the femoral head collapses and hip osteoarthritis develops (Shi et al., 2015; Zou et al., 2015; He et al., 2016).

Icariin is the main active ingredient of Epimedium, which has certain curative effects in the treatment of bone defects, osteoporosis, and osteonecrosis. Icariin can reduce the damage of glucocorticoids to bone microvascular endothelial cells (BMECs), improve the autophagy of BMECs induced by low concentrations of glucocorticoids, protect endothelial cells, and promote neovascularization (Wu et al., 2017; Yu et al., 2019; Zhang et al., 2019). ICA was found to have estrogen-like effects that directly promote the growth and maturation of osteoblasts. ICA can also increase osteogenic factors’ expression level and mineralization capacity to increase new bone formation (Wang et al., 2018) and can be used to regenerate bone defects and accelerate bone tissue repair (Zhang et al., 2017; Chen et al., 2019). At the same time, some studies have shown that ICA can inhibit bone loss and significantly improve osteoporosis (Liu et al., 2017; Tang et al., 2018). However, the effects of ICA on bone tissue morphology and structure in early ONFH pathological changes induced by glucocorticoids are less studied.

In this study, a rabbit model of ONFH induced by glucocorticoids was used for experiments, and the effects of traditional Chinese medicine Icariin on early bone tissue morphology, bone microstructure, and bone repair of ONFH were observed through PCI with synchrotron hard X-ray. Micro-CT scan reconstruction, pathological detection, and other methods were used for verification. Synchrotron radiation sources have the characteristics of high luminance, high collimation, and high spatial coherence (Bravin et al., 2013), which can overcome the shortcomings of X-ray absorption imaging methods. At the same time, the advantage of synchrotron radiation imaging technology is that it has extremely high spatial resolution and can realize the true reproduction of the ultrastructure inside the tissue (Sun et al., 2011). In this study, different bone tissue samples were used for tomographic scanning and 3D reconstruction imaging to observe the 3D structure of normal trabecular bone, trabecular bone after hormone injury, and trabecular bone after Icariin intervention. The results of the study were consistent with those of Micro-CT scan reconstruction and pathological slides. In the field of view of the unit of interest, the number of trabecular bones in the GC group was significantly reduced, the arrangement was sparse, the surface of the trabecular bone was rough, and the continuity was interrupted. The trabecular bone in the ICA group was relatively intact. It is worth noting that PCI with synchrotron hard X-ray technique is comparable to the performance of Micro-CT in showing the cartilage surface, subchondral bone plate, and trabecular bone spatial structure of the femoral head, etc. At the same time, this technique can offer 3D imaging results of bone tissue ultrastructure, and can clearly observe fine details such as the trabeculae thickness and surface smoothness. Therefore, it can be used as an effective alternative to HE staining to some extent.

This study has some limitations. Quantitative evaluation of PCI with synchrotron hard X-ray technique could not be done and thus, the verification of ONFH identified by PCI with synchrotron hard X-ray technique was only possible through Micro-CT scanning reconstruction and HE staining. Additionally, the role of ICA in a rabbit model of glucocorticoid-induced ONFH has only been studied and no further research has been conducted on its mechanism of action. Furthermore, the number of control animals in the rabbit model was small and hip imaging was not performed on all experimental animals before the start of the study, leading to a lack of baseline information.

## 5 Conclusion

The effects of Icariin were observed in an early glucocorticoid-induced ONFH rabbit model using PCI with synchrotron hard X-ray. Results revealed that Icariin weakens the destructive effect of glucocorticoids on bone tissue structure, improves bone tissue morphology, and stabilizes bone microstructure. These findings suggest that this technique may provide a definitive, non-invasive alternative to histological examination for the diagnosis of early ONFH.

## Data Availability

The original contributions presented in the study are included in the article/Supplementary Material, further inquiries can be directed to the corresponding authors.

## References

[B1] Aldridge J. M. 3rd Urbaniak J. R. (2004). Avascular necrosis of the femoral head: Etiology, pathophysiology, classification, and current treatment guidelines. Am. J. Orthop. (Belle Mead, N.J.) 33 (7), 327–332. 15344574

[B2] Betz O. Wegst U. Weide D. Heethoff M. Helfen L. Lee W. K. (2007). Imaging applications of synchrotron X-ray phase-contrast microtomography in biological morphology and biomaterials science. I. General aspects of the technique and its advantages in the analysis of millimetre-sized arthropod structure. J. Microsc. 227 (1), 51–71. 10.1111/j.1365-2818.2007.01785.x 17635659

[B3] Bravin A. Coan P. Suortti P. (2013). X-Ray phase-contrast imaging: From pre-clinical applications towards clinics. Phys. Med. Biol. 58 (1), R1–R35. 10.1088/0031-9155/58/1/R1 23220766

[B4] Calder J. D. Buttery L. Revell P. A. Pearse M. Polak J. M. (2004). Apoptosis--a significant cause of bone cell death in osteonecrosis of the femoral head. J. bone Jt. Surg. Br. 86 (8), 1209–1213. 10.1302/0301-620x.86b8.14834 15568539

[B5] Casey K. M. Gore F. Vilches-Moure J. G. Maruyama M. Goodman S. B. Yang Y. P. (2021). Management of morbidity and mortality in a New Zealand white rabbit model of SteroidInduced osteonecrosis of the femoral head. Comp. Med. 71 (1), 86–98. 10.30802/AALAS-CM-20-000071 33500020 PMC7898173

[B6] Chen M. Cui Y. Li H. Luan J. Zhou X. Han J. (2019). Icariin promotes the osteogenic action of BMP2 by activating the cAMP signaling pathway. Molecules 24 (21), 3875. 10.3390/molecules24213875 31661767 PMC6864436

[B7] Cohen-Rosenblum A. Cui Q. (2019). Osteonecrosis of the femoral head. Orthop. Clin. N. Am. 50 (2), 139–149. 10.1016/j.ocl.2018.10.001 30850073

[B8] Cooper M. S. (2004). Sensitivity of bone to glucocorticoids. Clin. Sci. Lond. Engl. 1979) 107 (2), 111–123. 10.1042/CS20040070 15113280

[B9] Cui Q. Jo W. L. Koo K. H. Cheng E. Y. Drescher W. Goodman S. B. (2021). ARCO consensus on the pathogenesis of non-traumatic osteonecrosis of the femoral head. J. Korean Med. Sci. 36 (10), e65. 10.3346/jkms.2021.36.e65 33724736 PMC7961868

[B10] Fukushima W. Fujioka M. Kubo T. Tamakoshi A. Nagai M. Hirota Y. (2010). Nationwide epidemiologic survey of idiopathic osteonecrosis of the femoral head. Clin. Orthop. Relat. Res. 468 (10), 2715–2724. 10.1007/s11999-010-1292-x 20224959 PMC2939331

[B11] Gao D. Pogany A. Stevenson A. W. Wilkins S. W. (1998). Phase-contrast radiography. Radiogr. a Rev. Publ. Radiological Soc. N. Am. Inc 18 (5), 1257–1267. 10.1148/radiographics.18.5.9747618 9747618

[B12] He M. Wang J. Wang G. Tian Y. Jiang L. Ren Z. (2016). Effect of glucocorticoids on osteoclast function in a mouse model of bone necrosis. Mol. Med. Rep. 14 (2), 1054–1060. 10.3892/mmr.2016.5368 27277157 PMC4940104

[B13] Johnston R. E. Washburn D. Pisano E. Burns C. Thomlinson W. C. Chapman L. D. (1996). Mammographic phantom studies with synchrotron radiation. Radiology 200 (3), 659–663. 10.1148/radiology.200.3.8756911 8756911

[B14] Li S. Lai Y. Zhou Y. Liao J. Zhang X. Zhang X. (2021). Pathogenesis of hormonal osteonecrosis of the femoral head and the target effect of related signaling pathways. Chin. J. Tissue Eng. Res. 25 (6), 935–941.

[B15] Li W. Huang Z. Tan B. Chen G. Li X. Xiong K. (2021). General recommendation for assessment and management on the risk of glucocorticoid-induced osteonecrosis in patients with COVID-19. J. Orthop. Transl. 31, 1–9. 10.1016/j.jot.2021.09.005 PMC8526281 34692412

[B16] Li Z. R. Cheng L. M. Wang K. Z. Yang N. P. Yang S. H. He W. (2018). Herbal Fufang Xian Ling Gu Bao prevents corticosteroid-induced osteonecrosis of the femoral head-A first multicentre, randomised, double-blind, placebo-controlled clinical trial. J. Orthop. Transl. 12, 36–44. 10.1016/j.jot.2017.11.001 PMC5866478 29662777

[B17] Liu H. Xiong Y. Zhu X. Gao H. Yin S. Wang J. (2017). Icariin improves osteoporosis, inhibits the expression of PPARγ, C/EBPα, FABP4 mRNA, N1ICD and jagged1 proteins, and increases Notch2 mRNA in ovariectomized rats. Exp. Ther. Med. 13 (4), 1360–1368. 10.3892/etm.2017.4128 28413478 PMC5377361

[B18] Liu W. Zhao Z. Na Y. Meng C. Wang J. Bai R. (2018). Dexamethasone-induced production of reactive oxygen species promotes apoptosis via endoplasmic reticulum stress and autophagy in MC3T3-E1 cells. Int. J. Mol. Med. 41 (4), 2028–2036. 10.3892/ijmm.2018.3412 29393368 PMC5810234

[B19] Mankin H. J. (1992). Nontraumatic necrosis of bone (osteonecrosis). N. Engl. J. Med. 326 (22), 1473–1479. 10.1056/NEJM199205283262206 1574093

[B20] Martin-Garcia J. M. Conrad C. E. Nelson G. Stander N. Zatsepin N. A. Zook J. (2017). Serial millisecond crystallography of membrane and soluble protein microcrystals using synchrotron radiation. IUCrJ 4 (4), 439–454. 10.1107/S205225251700570X PMC5571807 28875031

[B21] Meuli R. Hwu Y. Je J. H. Margaritondo G. (2004). Synchrotron radiation in radiology: Radiology techniques based on synchrotron sources. Eur. Radiol. 14 (9), 1550–1560. 10.1007/s00330-004-2361-x 15316744

[B22] Momose A. Fukuda J. (1995). Phase-contrast radiographs of nonstained rat cerebellar specimen. Med. Phys. 22 (4), 375–379. 10.1118/1.597472 7609717

[B23] Momose A. Takeda T. Itai Y. Hirano K. (1996). Phase-contrast X-ray computed tomography for observing biological soft tissues. Nat. Med. 2 (4), 473–475. 10.1038/nm0496-473 8597962

[B24] Mont M. A. Cherian J. J. Sierra R. J. Jones L. C. Lieberman J. R. (2015). Nontraumatic osteonecrosis of the femoral head: Where do we stand today? A ten-year update. J. bone Jt. Surg. Am. volume 97 (19), 1604–1627. 10.2106/JBJS.O.00071 26446969

[B25] Moya-Angeler J. Gianakos A. L. Villa J. C. Ni A. Lane J. M. (2015). Current concepts on osteonecrosis of the femoral head. World J. Orthop. 6 (8), 590–601. 10.5312/wjo.v6.i8.590 26396935 PMC4573503

[B26] Pfeiffer F. Bech M. Bunk O. Kraft P. Eikenberry E. F. Brönnimann C. (2008). Hard-X-ray dark-field imaging using a grating interferometer. Nat. Mater. 7 (2), 134–137. 10.1038/nmat2096 18204454

[B27] Pisano E. D. Johnston R. E. Chapman D. Geradts J. Iacocca M. V. Livasy C. A. (2000). Human breast cancer specimens: Diffraction-enhanced imaging with histologic correlation--improved conspicuity of lesion detail compared with digital radiography. Radiology 214 (3), 895–901. 10.1148/radiology.214.3.r00mr26895 10715065

[B28] Plotkin L. I. Manolagas S. C. Bellido T. (2007). Glucocorticoids induce osteocyte apoptosis by blocking focal adhesion kinase-mediated survival. Evidence for inside-out signaling leading to anoikis. J. Biol. Chem. 282 (33), 24120–24130. 10.1074/jbc.M611435200 17581824

[B29] Salomé M. Peyrin F. Cloetens P. Odet C. Laval-Jeantet A. M. Baruchel J. (1999). A synchrotron radiation microtomography system for the analysis of trabecular bone samples. Med. Phys. 26 (10), 2194–2204. 10.1118/1.598736 10535638

[B30] Seamon J. Keller T. Saleh J. Cui Q. (2012). The pathogenesis of nontraumatic osteonecrosis. Arthritis 2012, 601763. 10.1155/2012/601763 23243507 PMC3518945

[B31] Shah K. N. Racine J. Jones L. C. Aaron R. K. (2015). Pathophysiology and risk factors for osteonecrosis. Curr. Rev. Musculoskelet. Med. 8 (3), 201–209. 10.1007/s12178-015-9277-8 26142896 PMC4596210

[B32] Shi J. Wang L. Zhang H. Jie Q. Li X. Shi Q. (2015). Glucocorticoids: Dose-related effects on osteoclast formation and function via reactive oxygen species and autophagy. Bone 79, 222–232. 10.1016/j.bone.2015.06.014 26115910

[B33] Sun W. Li Z. R. Yang Y. R. Shi Z. C. Wang B. Liu B. (2011). Experimental study on phase-contrast imaging with synchrotron hard X-ray for repairing osteonecrosis of the femoral head. Orthopedics 34 (9), e530–e534. 10.3928/01477447-20110714-07 21902152

[B34] Sun W. Zhang Y. Gao F. Li Z. Li G. Pan L. (2015). Phase-contrast imaging with synchrotron hard X-ray of micro lesions of the cartilage of the femoral head in rabbits. Int. J. Clin. Exp. Med. 8 (11), 20086–20091. 26884921 PMC4723766

[B35] Takeda T. Momose A. Hirano K. Haraoka S. Watanabe T. Itai Y. (2000). Human carcinoma: Early experience with phase-contrast X-ray CT with synchrotron radiation--comparative specimen study with optical microscopy. Radiology 214 (1), 298–301. 10.1148/radiology.214.1.r00ja08298 10644140

[B36] Tang D. Ju C. Liu Y. Xu F. Wang Z. Wang D. (2018). Therapeutic effect of icariin combined with stem cells on postmenopausal osteoporosis in rats. J. bone mineral metabolism 36 (2), 180–188. 10.1007/s00774-017-0831-x 28681147

[B37] Wang D. Hu B. Hu C. Zhu F. Liu X. Zhang J. (2020). Clinical characteristics of 138 hospitalized patients with 2019 novel coronavirus-infected pneumonia in wuhan, China. Jama 323 (11), 1061–1069. 10.1001/jama.2020.1585 32031570 PMC7042881

[B38] Wang G. J. Cui Q. Balian G. (2000). The pathogenesis and prevention of steroid induced osteonecrosis. Clin. Orthop. Relat. Res. 370 (370), 295–310. 10.1097/00003086-200001000-00030 10660725

[B39] Wang X. Zheng L. Cao H. Qin L. (2019). Animal models and research on preventions and treatments of steroid-associated osteonecrosis of the femoral head. Chin. J. Orthop. 39 (23), 1462–1469.

[B40] Wang Z. Wang D. Yang D. Zhen W. Zhang J. Peng S. (2018). The effect of icariin on bone metabolism and its potential clinical application. Osteoporos. Int. a J. established as result Coop. between Eur. Found. Osteoporos. Natl. Osteoporos. Found. U. S. A. 29 (3), 535–544. 10.1007/s00198-017-4255-1 29110063

[B41] Weinstein R. S. Nicholas R. W. Manolagas S. C. (2000). Apoptosis of osteocytes in glucocorticoid-induced osteonecrosis of the hip. J. Clin. Endocrinol. Metab. 85 (8), 2907–2912. 10.1210/jcem.85.8.6714 10946902

[B42] Weiss P. Obadia L. Magne D. Bourges X. Rau C. Weitkamp T. (2003). Synchrotron X-ray microtomography (on a micron scale) provides three-dimensional imaging representation of bone ingrowth in calcium phosphate biomaterials. Biomaterials 24 (25), 4591–4601. 10.1016/s0142-9612(03)00335-1 12951002

[B43] Wu Y. Cao L. Xia L. Wu Q. Wang J. Wang X. (2017). Evaluation of osteogenesis and angiogenesis of icariin in local controlled release and systemic delivery for calvarial defect in ovariectomized rats. Sci. Rep. 7 (1), 5077. 10.1038/s41598-017-05392-z 28698566 PMC5505963

[B44] Xu J. Gong H. Lu S. Deasey M. J. Cui Q. (2018). Animal models of steroid-induced osteonecrosis of the femoral head-a comprehensive research review up to 2018. Int. Orthop. 42 (7), 1729–1737. 10.1007/s00264-018-3956-1 29705870

[B45] Youm Y. S. Lee S. Y. Lee S. H. (2010). Apoptosis in the osteonecrosis of the femoral head. Clin. Orthop. Surg. 2 (4), 250–255. 10.4055/cios.2010.2.4.250 21119943 PMC2981783

[B46] Yu H. Yue J. Wang W. Liu P. Zuo W. Guo W. (2019). Icariin promotes angiogenesis in glucocorticoid-induced osteonecrosis of femoral heads: *In vitro* and *in vivo* studies. J. Cell Mol. Med. 23 (11), 7320–7330. 10.1111/jcmm.14589 31507078 PMC6815836

[B47] Zhang Q. Gao F. Cheng L. Liu L. Sun W. Li Z. (2019). Effects of icariin on autophagy and exosome production of bone microvascular endothelial cells. Zhongguo Xiu Fu Chong Jian Wai Ke Za Zhi 33 (5), 568–577. 10.7507/1002-1892.201811009 31090350 PMC8337195

[B48] Zhang Y. Shen L. Mao Z. Wang N. Wang X. Huang X. (2017). Icariin enhances bone repair in rabbits with bone infection during post-infection treatment and prevents inhibition of osteoblasts by vancomycin. Front. Pharmacol. 8, 784. 10.3389/fphar.2017.00784 29163169 PMC5671559

[B49] Zou W. Yang S. Zhang T. Sun H. Wang Y. Xue H. (2015). Hypoxia enhances glucocorticoid-induced apoptosis and cell cycle arrest via the PI3K/Akt signaling pathway in osteoblastic cells. J. bone mineral metabolism 33 (6), 615–624. 10.1007/s00774-014-0627-1 25230819

