# Supplementary material for: Phase-contrast imaging with synchrotron hard X-ray reveals the effect of icariin on bone tissue morphology and microstructure in rabbits with early glucocorticoid-induced osteonecrosis of the femoral head
Source: Front Cell Dev Biol. 2023 May 4;11:1155532. doi: 10.3389/fcell.2023.1155532 (PMC10192577; doi:10.3389/fcell.2023.1155532)
Supplement: Supplementary file 1 [file Table1.DOCX]

Supplementary Material

Phase-contrast imaging with synchrotron hard X-ray reveals the effect of Icariin on bone tissue morphology and microstructure in rabbits with early glucocorticoid-induced osteonecrosis of the femoral head

Xu Yang^†^, Lijun Shi^†^, Aifeng Li^†^, Fuqiang Gao^*^, Wei Sun^*^, and Zirong Li

*** Correspondence:** Fuqiang Gao: [gaofuqiang@bjmu.edu.cn](mailto:gaofuqiang@bjmu.edu.cn) ; Wei Sun: [sunwei@zryhyy.com.cn](mailto:sunwei@zryhyy.com.cn)

# Supplementary Data

## Main experimental reagents and drugs

| Icariin | Solarbio | China |
| --- | --- | --- |
| Lipopolysaccharide (LPS) | Sigma | America |
| HE staining kit | Solarbio | China |
| Penicillin sodium | Solarbio | China |
| Formaldehyde | Solarbio | China |
| Ethyl carbamate | Solarbio | China |
| Methylprednisolone (MPS) | Pfizer Inc. | America |

## Main instruments and equipment

| Hard tissue slicer | Leica | Germany |
| --- | --- | --- |
| Layered dehydrator | Leica | Germany |
| paraffin embedding station | Scilight-Peptide | China |
| Inverted phase contrast microscope | Nikon | Japan |
| Micro-CT | General | America |
| Mimics software | Materialise | Belgium |

## Micro-CT scanning

The left femoral head specimen was fixed in (40 g/L) 10% paraformaldehyde fixative for 48 hours, replaced by ethanol immersion with a volume fraction of 75%, and replaced by saline immersion the day before the scan. The femoral head specimen was placed in the Micro-CT scanning coil for scanning. A CT spiral scan was performed starting from the distal end of the femoral head specimen, with one tomographic section every 18 µm. The scanning parameters were as follows: tube voltage of 80 kV, tube current of 450 µA, spatial resolution of 45 µm × 45 µm × 45 µm, and exposure time of 2000 ms. The obtained CT data were imported into Mimics software in DICOM format, and the bony structure of the femoral head was reconstructed in three dimensions by creating templates, threshold segmentation, and smear filling. Cross-sectional, coronal, sagittal, and three-dimensional views were selected to observe the three-dimensional structure of the femoral head specimen from multiple angles.

## HE staining

The specimen of the left femoral head was dissected longitudinally along the coronal plane with a hard tissue slicer after Micro-CT scanning, and then fixed in 10% neutral formaldehyde for 7 days and decalcified by immersion in 5% nitric acid solution for 3 days. After satisfactory decalcification, the specimens were dehydrated with gradient ethanol in a stratified dehydrator, embedded in conventional paraffin, cut, smoothed, and polished by a microtome, and 15 µm thick sections were prepared. After dewaxing, hematoxylin and eosin (HE) staining was performed to observe the changes in bone trabecular structure, bone cells, and bone marrow fat morphology under the light microscope.
